# Prediction model based on gut microbiota as a non-invasive tool for gastric cancer diagnosis

**DOI:** 10.1007/s00253-025-13548-5

**Published:** 2025-07-10

**Authors:** Changchang Chen, Chen Chen, Xiaoguang Zheng, Weizhong Wang, Jian Shen, Gulei Jin, Jianxin Lyu, Lijun Lin

**Affiliations:** 1 Laboratory Medicine Center, Department of Clinical Laboratory, Zhejiang Provincial People’s Hospital (Affiliated People’s Hospital), Hangzhou Medical College, Hangzhou, Zhejiang China; 2 School of Laboratory Medicine, Hangzhou Medical College, Hangzhou, Zhejiang China; 3 Laboratory Medicine Center, Department of Transfusion Medicine, Zhejiang Provincial People’s Hospital (Affiliated People’s Hospital), Hangzhou Medical College, Hangzhou, Zhejiang China; 4 Hangzhou Guhe Information and Technology Company, Hangzhou, Zhejiang China; 5 School of Basic Medicine and Forensic Medicine, Hangzhou Medical College, Hangzhou, Zhejiang China

**Keywords:** Gastric cancer, 16S rRNA, Gut microbiota, Non-invasive diagnosis, Tumor biomarkers

## Abstract

**Abstract:**

Gastric cancer (GC) is a malignant cancer of the digestive tract with high morbidity and mortality. Previous studies have shown that current diagnostic methods largely rely on invasive procedures. Moreover, there are no highly sensitive and accurate biomarkers available for early GC diagnosis. Recent studies using 16S rRNA technology show that gut microbiota can differentiate between diseased and healthy individuals. However, fewer studies emphasize the gut microbiome’s value in GC diagnosis. In this study, we collected 455 fecal samples, including 100 from healthy individuals (healthy controls [HCs]), 153 from GC patients, 43 from patients with non-neoplastic diseases of the stomach, and 159 from verification individuals. Our analysis revealed a significantly increased microbial richness in the GC group (Chao1 index, *P* < 0.05) and distinct compositional differences (principal coordinates analysis). Linear discriminant analysis effect size analysis identified 19 HC-enriched genera (e.g., *Bacteroides*) and 31 GC-enriched genera (e.g., *Streptococcus*). The random forest model selected 20 key diagnostic genera, achieving an area under the receiver operating characteristic curve (AUC) of 0.81. By integrating 10 tumor biomarkers, the combined diagnostic model improved the AUC to 0.86 (validation set: 0.84). Tumor biomarker positivity (60.78%) did not directly correlate with microbiota, but the microbiota-biomarker model improved non-invasive diagnostic accuracy, providing a new approach for early GC screening.

**Key points:**

• *Changchang Chen and Chen Chen contributed equally to this work*

• *Gut microbiota changes significantly in gastric cancer*

• *Microbiome shows promise as non-invasive diagnostic markers*

• *The combined microbiota-tumor marker model improves diagnosis*

## Introduction

Gastric cancer (GC) is a significant global health concern, with 1,103,701 new cases and 782,785 deaths reported in 2018 (Wong et al. 2021). Genetic factors, *Helicobacter pylori* infection, smoking, alcohol intake, and eating habits are the main risk factors for GC (Rawla and Barsouk 2019). Although GC incidence has decreased with reduced smoking and declining *H. pylori* prevalence, it continues to cause substantial mortality in low-resource regions (Lin et al. 2021). The early diagnosis of GC remains challenging due to subtle clinical symptoms and the necessity of invasive testing. The current clinical discovery and diagnosis of GC primarily rely on gastroscopy and biopsy.

Tumor biomarkers are biochemical substances that can be detected in blood or body fluids, released by tumor cells or their corresponding cells within the body (Nagpal et al. 2016). They are also routinely used as a method of early tumor screening in physical examinations and possible tumor patients. Currently widely used serum tumor markers for GC, such as carcinoembryonic antigen (CEA), cancer antigen 125 (CA125), and carbohydrate antigen 19–9 (CA19-9), are glycoproteins (proteins with attached carbohydrate chains) that may be elevated in GC patients, but due to their low sensitivity and specificity, their diagnostic utility is limited (Xia and Aadam 2022). Additionally, the recently proposed markers for screening high-risk GC patients, serum pepsinogen II and gastrin-17, lack sensitivity and specificity, and thus do not provide any diagnostic value (Gašenko et al. 2023). According to the research, the positive rate of tumor markers in patients with GC is generally low, and the positive rate of individual tumor markers is generally less than 30% (Shimada et al. 2014). The detection rate of GC by simultaneous detection of multiple tumor markers is also limited (Nagpal et al. 2016). These factors ultimately result in the majority of patients receiving diagnoses at advanced stages, with concomitantly limited treatment options (Necula et al. 2019). The absence of non-invasive diagnostic techniques often leads patients to undergo endoscopy only after experiencing pronounced symptoms, thereby exacerbating the prevalence of late-stage diagnoses (Van Cutsem et al. 2016). Besides, the burden of GC remains significant in most countries (Thrift and El-Serag 2020).

Approximately 10^14^ populations of microbiome colonize in human gut intestine mucosa (Zhang et al. 2015), including bacteria, fungi, and viruses (Dicks et al. 2018). The intestinal microbiome performs beneficial roles in various domains, including digestion and substance synthesis (Hartmann et al. 2019), but microbiota dysregulation may happen when the microbial composition of the microflora has changed (Thursby and Juge 2017). Recent studies have shown that microbiota dysregulation can lead to several diseases, such as irritable bowel syndrome (Halkjær et al. 2018), inflammatory bowel disease (Lehmann et al. 2019), liver cirrhosis (Bajaj et al. 2018), and gastrointestinal malignancies (Gunathilake et al. 2019). Several studies have demonstrated that the gut microbiome is correlated with GC (Qi et al. 2019; Wu et al. 2020). Furthermore, our prior research demonstrated that the gut microbiota in GC patients significantly differs from that of healthy individuals, with distinct microbiomes present during both early and advanced stages of GC (Chen et al. 2021, 2022a, 2022b; Zhang et al. 2021). However, a reliable and validated gut microbiome model for GC screening is unavailable. Therefore, this study integrated specific gut microbiotas with clinical tumor biomarkers to develop a joint prediction model for diagnosing GC.

## Material and methods

### Study population

A total of 455 individuals were recruited from Zhejiang Provincial People’s Hospital between April 2018 and January 2023, including 153 GC patients, 100 healthy controls (HCs), 43 gastric disease controls (GDCs, including chronic atrophic gastritis, gastric ulcers, gastric polyps, etc.), and 159 verification individuals. Among them, part of the fecal data came from our previous studies (Chen et al. 2022a, 2022b; Zhang et al. 2021). All the included patients with GC were first-time patients who had not received any treatment and were confirmed using pathological examinations or gastroscopy. Healthy individuals were recruited by the Department of Health Examination Center of Zhejiang Provincial People’s Hospital. All patients provided fecal and blood samples. Additionally, participants’ medical data were collected by reviewing their clinical records.

Patients’ information included gender, age, and TNM stage (“T” denotes the primary tumor’s size and extent; “N” indicates whether cancer has spread to nearby lymph nodes; and “M” signifies whether the cancer has metastasized), as shown in Table 1. Fecal samples were collected from each individual and stored at −80 °C until 16S rRNA sequencing was performed.This study was approved by the Ethics Committee of Zhejiang Provincial People’s Hospital (No. 2022QT010). All samples were anonymously given.

**Table 1:** Clinical features of enrolled participants

Characteristics	Gastric cancer	Health	Non-neoplastic diseases of the stomach	Verification individuals
Sex	153	100	43	159
Male	116 (75.82%)	64 (64.00%)	22 (51.16%)	93 (58.49%)
Female	37 (24.82%)	36 (36.00%)	21 (48.84%)	66 (41.51%)
Age (years)	64.70 ± 12.07	55.16 ± 1.33	62.02 ± 1.41	60.08 ± 12.79
Tumor stage				
I	51 (33.33%)	-	-	
II	21 (13.73%)	-	-	
III, IV	67 (43.79%)	-	-	
Unknown	14 (9.15%)	-	-	

Measurement data are expressed as mean ± standard error of the mean (SEM), and quantitative data are expressed as number (percentage, %)

## Tumor biomarkers test

The following biomarkers were analyzed: cytokeratin 19 fragment (CYFRA21-1), cancer antigen 15–3 (CA15-3), cancer antigen 72–4 (CA72-4), neuron-specific enolase (NSE), CEA, CA125, pro-gastrin-releasing peptide (ProGRP), alpha-fetoprotein (AFP), squamous cell carcinoma antigen (SCCA), and CA19-9. Furthermore, blood samples were collected using inert gel anticoagulation tube and examined through an automatic chemiluminescence immunoanalyzer (Roche, Cobas 8000, Basel, Switzerland). Ten tumor biomarkers were measured using their specific antigen kits (Roche, Basel, Switzerland). All assays were performed strictly according to the manufacturer’s instructions. Additionally, the reference ranges provided by the manufacturer for each assay were used for clinical interpretation (Results).

## DNA extraction and (polymerase chain reaction) PCR amplification

The microbial genomic DNA was extracted using the Guhe Stool Mag DNA Kit (Guhe Info Technology Co., Ltd, Zhejiang, China). The quantity and quality of extracted DNAs were tested using a NanoDrop ND-2000 spectrophotometer (Thermo Fisher Scientific, Waltham, MA, USA) and agarose gel electrophoresis, respectively. PCR amplification of the V4 region of 16S rRNA was performed using the primer 515 F (5’-GTGCCAGCMGCCGCGGTAA-3’) and 806R (5’-GGACTACHVGGGTWTCTAAT-3’). Specific 6-bp barcodes were synthesized into sequences for multiplex sequencing. The PCR mixture contained 25 μL of Phusion High-Fidelity PCR Master Mix, 3 μL (10 uM) of primer, 10 μL of DNA template, 3μL of dimethyl sulfoxide (DMSO), and 6 μL of ddH_2_O. PCR cycling procedures were as follows: initial denaturation at 98 °C for 30 s, followed by 25 cycles consisting of denaturation at 98 °C for 15 s, annealing at 58 °C for 15 s, and extension at 72 °C for 15 s, with a final extension of 1 min at 72 °C. The amplified PCR were purified using Agencourt AMPure XP Beads (Beckman Coulter, Indianapolis, IN, USA) and quantified by PicoGreen dsDNA Assay Kit (Invitrogen, Carlsbad, CA, USA). Amplicons were pooled after quantification, and paired-end 2 × 150 bp sequencing was performed using the Illumina NovaSeq6000 platform (Illumina, San Diego, CA, USA) at GUHE Info Technology Co., Ltd (Hangzhou, China).

## Sequence analysis

Raw paired-end sequencing reads were initially processed to assign reads to their respective samples based on perfect barcode matches. Paired-end reads were then merged using Vsearch v2.22.1 with the command fastq_mergepairs and requiring a minimum overlap of 5 bp (–fastq_minovlen 5). Amplicon sequence variants (ASVs) were subsequently generated through dereplication (derep_fulllength), quality filtering and denoising using the UNOISE3 algorithm (cluster_unoise), and chimera removal using uchime3_denovo within Vsearch. Finally, reads were mapped back to the generated ASVs using usearch_global with a 100% identity threshold (–id 1.0) to create an ASV abundance table.

To account for variability in sequencing depth, the raw ASV count table was normalized per sample to a total sum of 100,000 reads using a custom Perl script, resulting in a normalized abundance table. A representative sequence was selected for each ASV using default parameters in Vsearch. Both the normalized ASV table and the representative sequences were imported into QIIME 2 (https://qiime2.org). ASVs with a total frequency across all samples below 10 counts in the normalized table were removed using qiime feature-table filter-features (–p-min-frequency 10) to yield the final filtered ASV table for downstream analyses.

## Taxonomic assignment

Taxonomic classification was assigned to the ASV representative sequences in QIIME 2 using a pre-trained Naïve Bayes classifier (qiime feature-classifier classify-sklearn). The classifier was trained on the SILVA database (https://www.arb-silva.de) (release 138, 99% operational taxonomic units (OTU) identity) and trimmed to the V4 region, amplified by the 515F/806R primers. ASVs unclassified at a specific taxonomic level were excluded from analyses performed at that level.

## Microbiome diversity analysis

A phylogenetic tree was constructed from the representative sequences using qiime phylogeny align-to-tree-mafft-fasttree. Alpha and beta diversity metrics were calculated using qiime diversity core-metrics-phylogenetic based on the filtered, normalized ASV table. This step involved applying a rarefaction depth of 10,000 sequences per sample (–p-sampling-depth 10000).

Alpha diversity was assessed using Observed Features (ASV richness), the Shannon index (reflecting both richness and evenness), and the Simpson index (reflecting dominance). Chao1, ACE, and Good's coverage metrics were also calculated separately using qiime diversity alpha. Furthermore, group differences in alpha diversity indices were statistically evaluated using the Kruskal–Wallis test.

Beta diversity was evaluated using Bray–Curtis dissimilarity, weighted UniFrac distance, and unweighted UniFrac distance. Differences in community structure between groups were visualized using principal coordinates analysis (PCoA). Statistical significance of group clustering was tested using analysis of similarities (ANOSIM) and permutational multivariate analysis of variance (PERMANOVA) with 999 permutations, implemented in the R package “vegan” (https://cran.r-project.org/web/packages/vegan/index.html). Principal component analysis (PCA) based on ASV relative abundance profiles was also employed for visualization.

## Statistical analysis for differential abundance and alassification

Differential abundance of taxa at phylum, class, order, family, genus, and species levels between groups was assessed using the non-parametric Kruskal–Wallis test (for multi-group comparisons) or Wilcoxon rank-sum test (for two-group comparisons), implemented in the R 4.3.1 (https://www.R-project.org/). Significant differences were visualized using box-and-whisker plots. Additionally, linear discriminant analysis effect size (LEfSe) analysis was used to identify statistically significant and biologically relevant biomarkers between groups, using default parameters (linear discriminate analysis (LDA) score > 2.0, *P* < 0.05).

To evaluate the potential of the gut microbiome to distinguish between groups, Random Forest classification models were constructed using the R package randomForest. Models were built using the relative abundances of ASVs from the filtered, normalized table, with 1,000 trees (ntree = 1000) and default settings for other parameters (e.g., mtry). Model performance and generalization error were estimated using tenfold cross-validation.

## Results

### Comparation of gut microbiome in GC patients and HCs

**Fig. 1:**
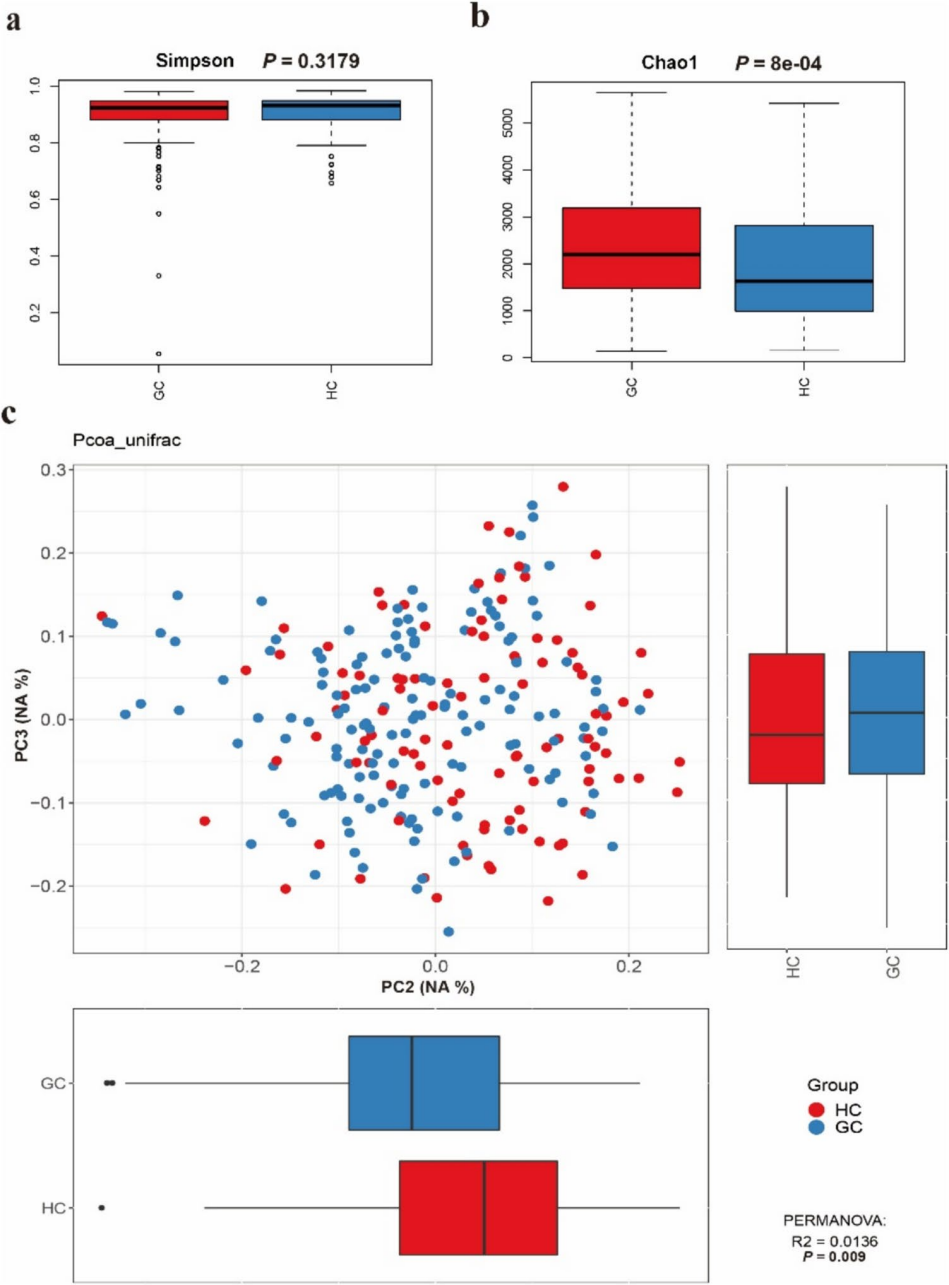
Comparison of gut microbiota between gastric cancer (GC) patients and healthy controls (HCs). (**a**) Simpson and (**b**) Chao1 indices showed no significant difference in microbial diversity but increased richness in GC patients. (**c**) Principal coordinates analysis at the genus level revealed significant compositional differences between the GC and HC groups (NA: This principal component shows no significant difference)

To acquire the influence of tumor biomarkers on gut microbiome of GC patients, we compared gut microbiota of the GC and HC groups. The Simpson and Chao1 indices were used to describe the diversity and abundance of gut microbiota in these two groups (Fig. 1a and b). Then, PCoA based on the genus level was used to value the similarity between these two groups (Fig. 1c). The Simpson and Chao1 indices indicated no significant difference in microbial diversity between the GC and HC groups, but microbiota richness is significantly higher in the GC group. Additionally, the PCoA results showed a significant statistical difference in microbiota composition between the GC and HC groups.

**Fig. 2:**
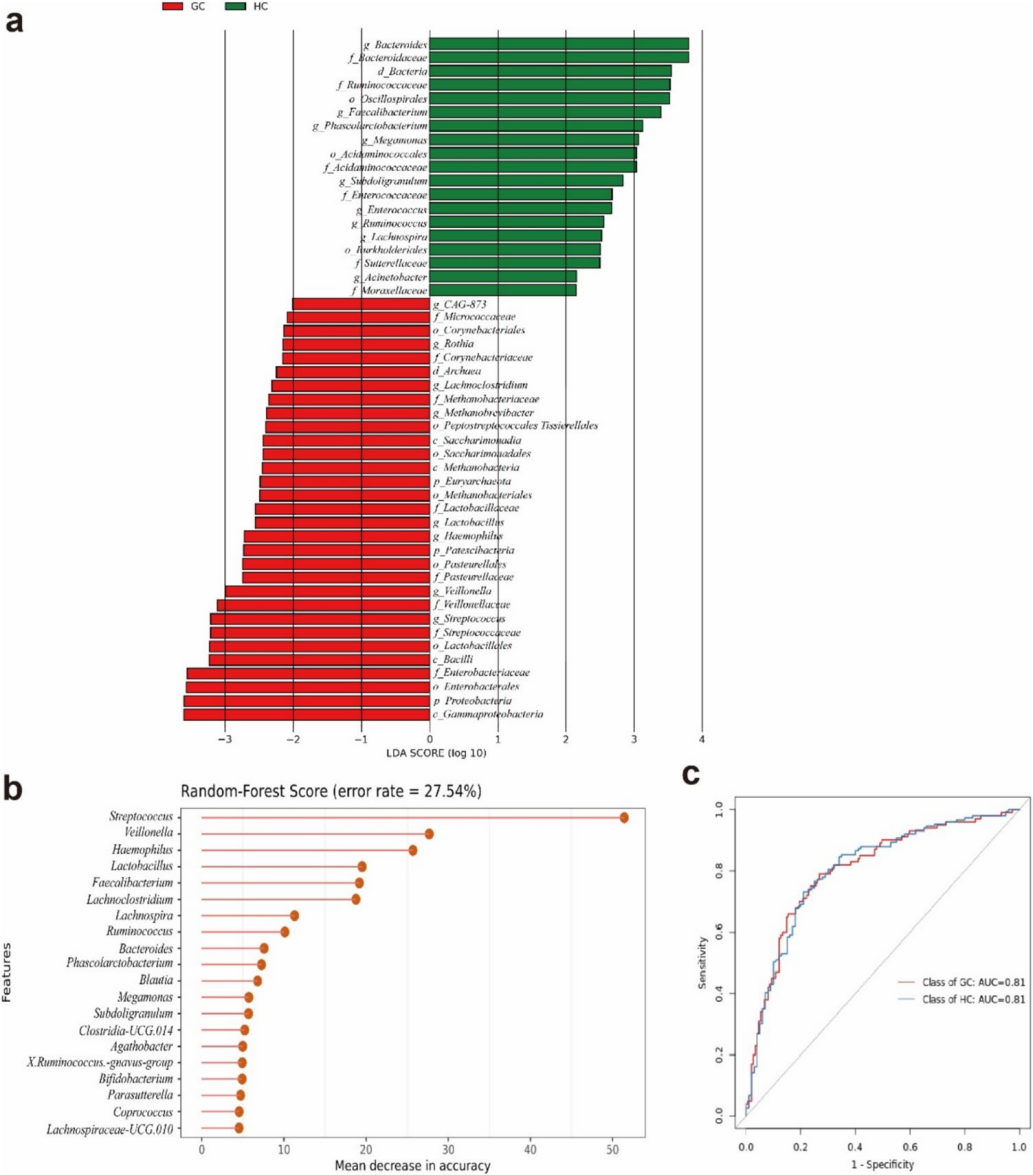
Differentially abundant gut microbiota and diagnostic potential. (**a**) Linear discriminant analysis effect size analysis showed 19 microbiotas enriched in HC (green) and 31 taxa more abundant in GC (red). (**b**) Random forest analysis listed 20 key genera for distinguishing GC patients, including *Streptococcus*, *Veillonella*, and *Lactobacillus*. The classification model had an error rate of 27.54%. (**c**) Receiver operating characteristic analysis shows an area under the curve of 0.81, indicating good diagnostic efficacy for gut microbiota in distinguishing between GC and HC groups

Additionally, LEfSe analysis was used to identify gut microbiome that exhibited significant difference in expression between the GC and HC groups (Fig. 2a). The results showed the enrichment of 19 gut microbiotas in the HC group (in green), including *Bacteroides, Bacteroidaceae, Bacteria, Ruminococcaceae Oscillospirales, Faecalibacterium, Phascolarctobacterium, Megamonas, Acidaminococcales, Acidaminococcaeae, Subdoligranulum, Enterococcaceae, Enterococcus, Ruminococcus, Lachnospira, Burkholderiales, Sutterellaceae, Acinetobacter, and Moraxellaceae*. Besides, the results showed the enrichment of 31 gut microbiotas in the GC group (in red), including *CAG_873*, *Micrococcaceae*, *Corynebacteriales*, *Rothia*, *Corynebacteriaceae*, *Archaea*, *Lachnoclostridium*, *Methanobacteriaceae*, *Methanobrevibacter*, *Peptostreptococcales_Tissierellales, Saccharimonadia*, *Saccharimonadales*, *Methanobacteria*, *Euryarchaeota*, *Methanobacteriales*, *Lactobacillaceae*, *Lactobacillus*, *Haemophilus*, *Patescibacteria*, *Pasteurellales*, *Pasteurellaceae*, *Veillonella*, *Veillonellaceae*, *Streptococcus*, *Streptococcaceae*, *Lactobacillales*, *Bacilli*, *Enterobacteriaceae*, *Enterobacterales*, *Proteobacteria*, and *Gammaproteobacteria*.

Furthermore, the random-forest analysis was used to value specific gut microbiota in differentiating GC patients. At the genus level, from top to bottom, 20 genus microbiomes were listed (Fig. 2b), including *Streptococcus*, *Veillonella*, *Haemophilus*, *Lactobacillus*, *Faecalibacterium*, *Lachnoclostridium*, *Lachnospira*, *Ruminococcus*, *Bacteroides*, *Phascolarctobacterium*, *Blautia*, *Megamonas*, *Subdoligranulum*, *Clostridia_UCG.014*, *Agathobacter*, *X.Ruminococcus_gnavus_group*, *Bifidobacterium*, *Parasutterella*, *Coprococcus*, and *Lachnospiraceae_UCG.010*. The error rate was used to predict the classification model with the characteristic microbiotas. Additionally, the rate was 27.54%. The diagnostic efficacy of gut microbiota was evaluated using the receiver operating characteristic (ROC) analysis, yielding an area under the curve (AUC) value of 0.81 for the GC group compared to the HC group (Fig. 2c).

## Difference of gut microbiota in GC patients with positive or negative tumor biomarkers

Ultimately, we established a GC gut microbiota prediction model based on gut microbiota data in conjunction with some tumor marker data collected from our clinical GC patients (Table 2), including CEA, CA19-9, AFP, CA12-5, CA15-3, CA72-4, CYFRA 21–1, SCCA, NSE, and ProGRP. The reference ranges were as follows: CYFR21-1, 0–3.8 ng/mL; CA15-3, 0–28.5 ng/mL; CA72-4, 0–19.3 U/mL; NSE, 0–20 ng/mL; CEA, 0–5 μg/L; CA125, 0–35 U/mL; ProGRP, 25–78 pg/mL; AFP, 0–20 μg/L; SCC, 0–3 ng/mL; CA19-9, 0–37 μ/mL. The positive rate of tumor biomarkers in GC was 60.78%.

**Table 2:** Serum expression levels of tumor markers and survival in all sample sets

Characteristics	Gastric cancer patients	Non-neoplastic individuals
Sex	153	107
Male	116 (75.82%)	72 (67.29%)
Female	37 (24.18%)	35 (32.71%)
Age (years)	64.70 ± 12.07	59.92 ± 11.53
CYFR21-1		
Positive	48 (31.37%)	13 (12.15%)
Negative	105 (68.63%)	94 (87.85%)
CA15-3		
Positive	4 (2.61%)	1 (0.93%)
Negative	149 (97.39%)	106 (99.07%)
CA72-4		
Positive	16 (10.46%)	4 (3.74%)
Negative	137 (89.54%)	103 (96.26%)
NSE		
Positive	6 (3.92%)	1 (0.93%)
Negative	147 (96.08%)	106 (99.07%)
CEA		
Positive	34 (22.22%)	4 (3.74%)
Negative	119 (77.88%)	103 (96.26%)
CA125		
Positive	24 (15.69%)	3 (2.80%)
Negative	129 (94.31%/	104 (97.20%)
ProGRP		
Positive	16 (10.46%)	3 (2.80%)
Negative	137 (89.54%)	104 (97.20%)
AFP		
Positive	5 (3.27%)	0 (0%)
Negative	148 (63.73%)	107 (100.00%)
SCC		
Positive	9 (5.88%)	0 (0%)
Negative	144 (94.12%)	107 (100.00%)
CA199		
Positive	28 (18.30%)	0 (0%)
Negative	125 (81.70%)	107 (100.00%)
Total positive rates	93 (60.78%)	32 (29.91%)
Total negative rates	60 (39.22%)	75 (30.09%)

Measurement data are expressed as mean ± SEM, and quantitative data are expressed as number (percentage, %)

**Fig. 3:**
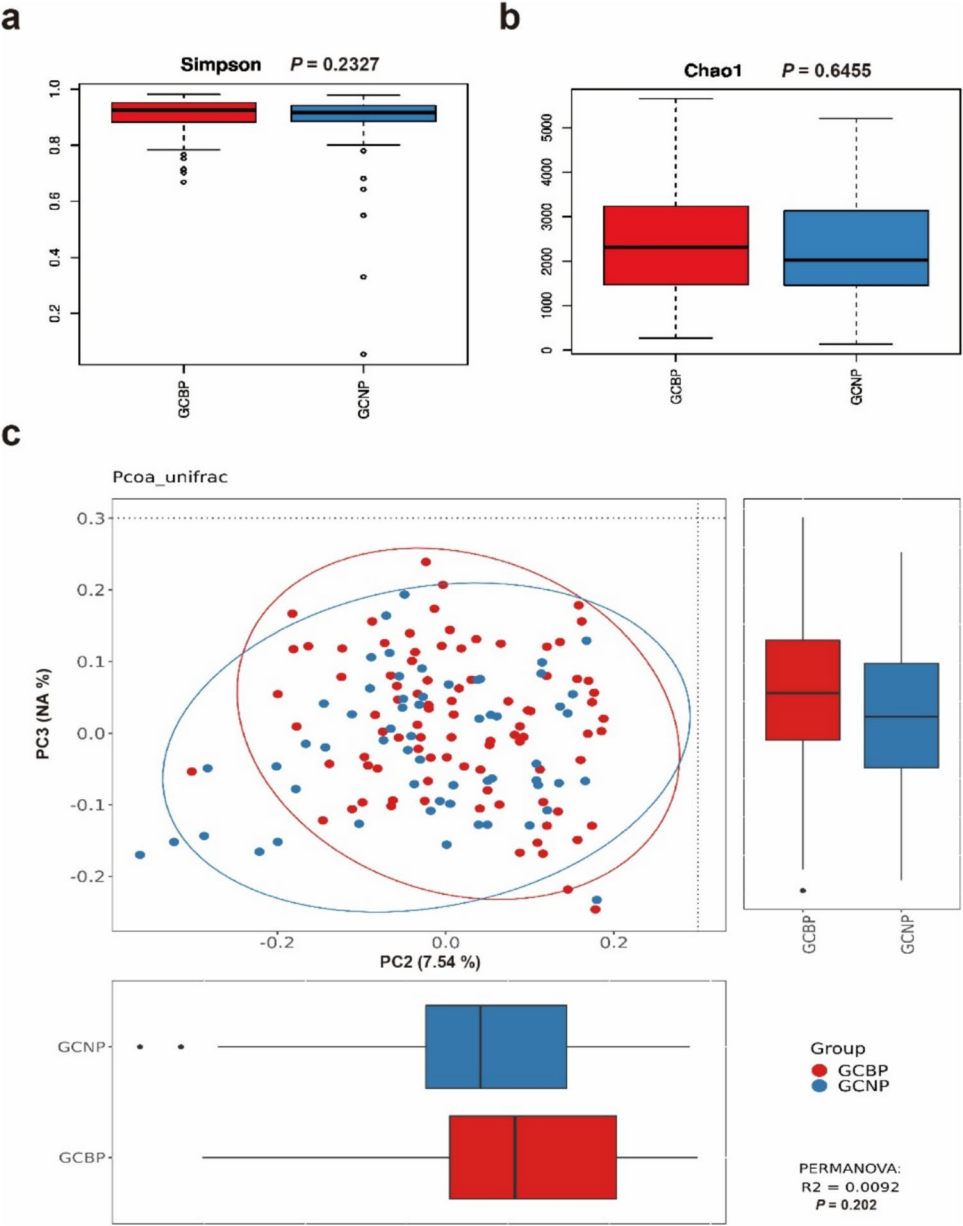
Correlation between tumor biomarker expression and gut microbiota in gastric cancer (GC) patients. (**a**) Simpson and (**b**) Chao1 indices showed no significant difference in gut microbiome diversity and richness between GC patients with positive tumor markers (GCBP) and those with negative tumor markers (GCNP) (*P* = 0.2327 and *P* = 0.6455, respectively). (**c**) Principal coordinates analysis reveals no compositional differences between GCBP and GCNP groups (*P* = 0.194). Linear discriminant analysis effect size analysis also did not identify any specific microbiota distinguishing GCBP from GCNP (NA: This principal component shows no significant difference)

To investigate the correlation between tumor biomarker expression and gut microbiota of GC patients, two indicators, the Simpson and Chao1 indices (Fig. 3a and b) were used to evaluate the diversity and richness of gut microbiomes in GC patients with positive tumor markers (GCBP) and GC patients with negative tumor markers (GCNP). The results showed no difference between GCBP and GCNP in the diversity and abundance of gut microbiomes (*P* = 0.2327 and *P* = 0.6455, respectively). The composition of gut microbiomes between GCBP and GCNP did not show any differences (*P* = 0.194) (Fig. 3c). LEfSe analysis was also used to distinguish specific gut microbiomes between these two groups; however, no gut microbiota could be found in this analysis.

## Prediction model of gut microbiota to distinguish GC from HC and other digestive tract diseases

**Fig. 4:**
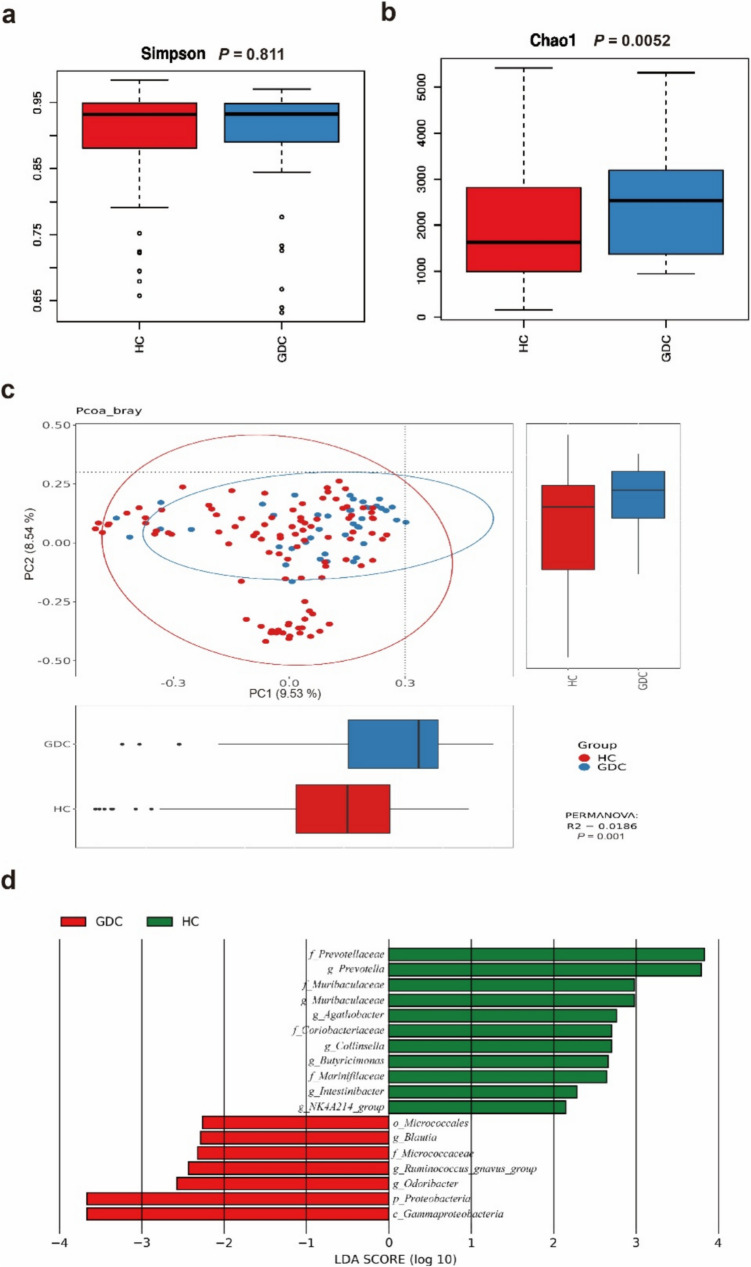
Evaluation of gut microbiota in gastric cancer (GDC) and healthy controls (HCs). (**a**) Simpson index indicated no significant difference in microbial diversity between the GDC and HC groups. (**b**) Chao1 index showed increased microbiota richness in the GDC group. (**c**) Principal coordinates analysis revealed significant compositional differences between the GDC and HC groups. (**d**) LEfSe analysis identified differential microbiota, with 11 taxa enriched in the HC (green) and 7 taxa in the GDC group (red). No overlapping specific microbiota were found between these groups

To enhance the precision of the gut microbiota model in identifying GC, we performed microbiota analysis on patients from both the HC group and GDC groups. The Simpson index indicates no significant difference in microbial diversity between the GDC and HC groups (Fig. 4a). However, the Chao1 index showed a significant increase in the microbiota richness in the GDC group (Fig. 4b). Furthermore, the results of PCoA demonstrated a statistically significant difference in microbiota composition between the two groups (Fig. 4c).

LEfSe analysis was employed to identify differential gut microbiota between the GDC and HC groups to mitigate the potential for false positives that might arise from the model developed using gastric cancer patients and healthy population controls (Fig. 4d). The results indicated that a total of 11 bacteria taxa were enriched in the HC group (in green), including the family level: *Prevotellaceae, Muribaculaceae, Coriobacteriaceae, and Marinifilaceae;* and the genus level: *Prevotella, Muribaculaceae, Agathobacter, Collinsella, Butyricimonas, Intestinibacter*, and *NK4A214_group*. In the GDC group, seven bacteria taxa were found to be enriched (in red), comprising *Microccales* (order level), *Blautia* (genus level), *Micrococcaceae* (family level), *Ruminococcus_gnavus_group* (genus level), *Odoribacter*(genus level), *Proteobacteria* (phylum level), *Gammaproteobacteria* (class level). Upon further comparison with the GDC group, no overlapping specific microbiota were identified between the two groups, thus not affecting the establishment of the predictive model.

## Diagnostic value analysis of various models and application value of the comprehensive prediction model

**Fig. 5:**
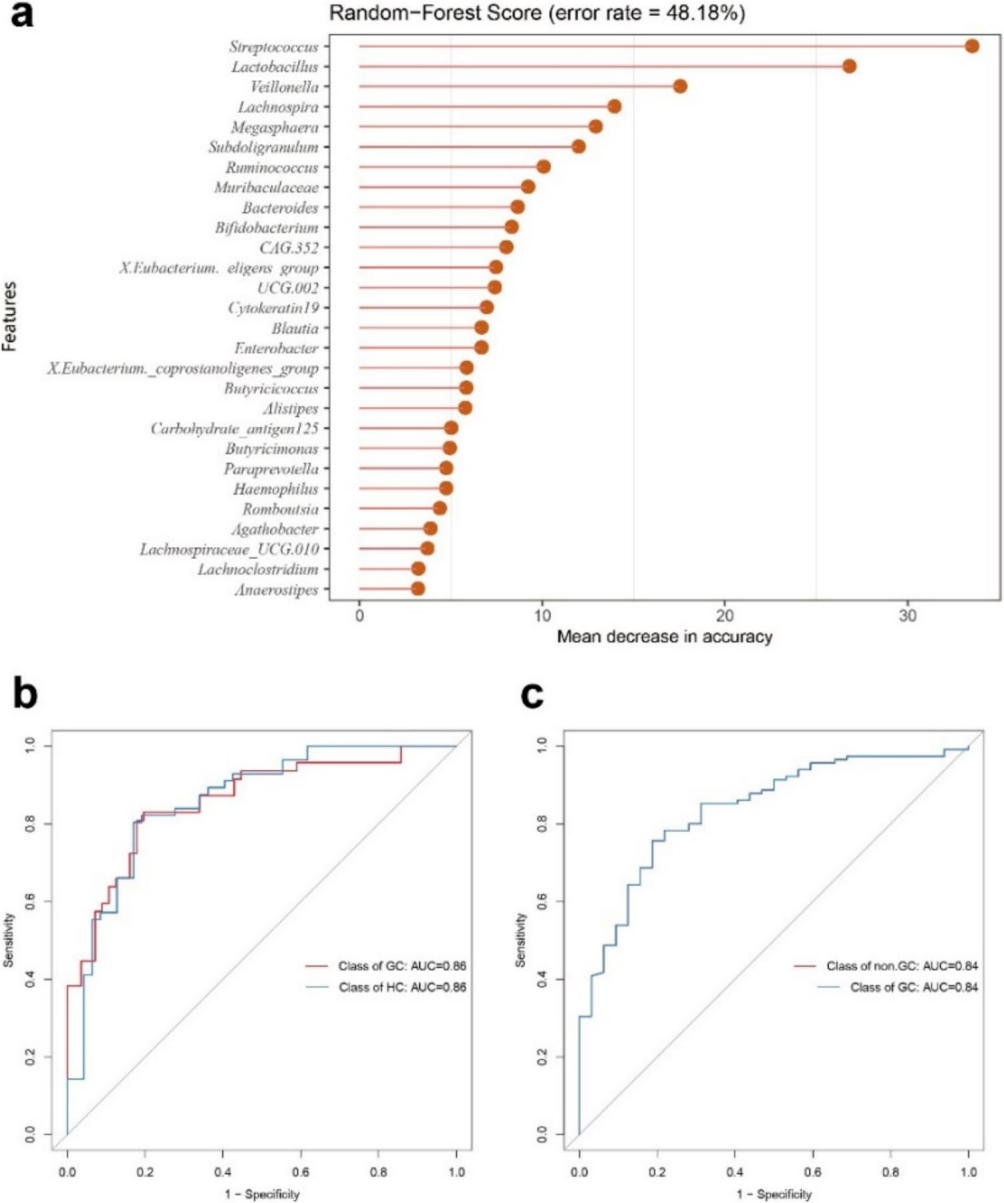
Diagnostic model integration and validation. (**a**) Construction of a predictive model integrating gut microbiota with tumor markers for gastric cancer. (**b**) Diagnostic efficacy of the model had an AUC of 0.86. (**c**) Independent validation of the model yielded an AUC of 0.84

We integrated gut microbiota with tumor markers to construct the GC gut microbiota predictive model (Fig. 5a) and assessed its diagnostic efficacy (Fig. 5b). Additionally, the model was independently validated in the validation set (Fig. 5c), with AUC values of 0.86 and 0.84, respectively.

## Discussion

The findings of this study underscore the potential of gut microbiota as non-invasive biomarkers for the diagnosis of GC. Our comprehensive analysis of gut microbiota composition in GC patients compared to HCs revealed distinct microbial profiles, highlighting the potential diagnostic utility of these differences while controlling for other gastric diseases. Furthermore, the integration of gut microbiota data with traditional tumor biomarkers significantly improved diagnostic accuracy, suggesting a novel and promising approach for early GC detection.

## Alterations in gut microbiota composition in GC patients

Although the exact mechanism of GC remains unclear, diet appears to be a significant factor contributing to its occurrence (Bouras et al. 2022). Additionally, GC is associated with factors such as environmental influences, *H. pylori* infection, and smoking (Thrift et al. 2023). Several studies have proved that diet can rapidly and reproducibly alter the human gut microbiome (David et al. 2014), which is associated with cell damage and changes in the tumor immune microenvironment through direct or extra-intestinal effects, facilitating the occurrence and development of gastrointestinal tumors (Tong et al. 2021). In this context, we explored the relationship between gut microbiome and GC patients.

We collected and analyzed gut microbiome diversity and richness between the GC and HC groups. The results showed notable alterations in these groups. This finding is highly consistent with our previous research and aligns with the results obtained by other scholars (Qi et al. 2019; Zhang et al. 2021). Specifically, the Simpson and Chao1 indices indicated that while microbial diversity did not differ significantly between the two groups, there was a marked increase in microbial richness in the GC group (Fig. 1a and b). PCoA further confirmed significant compositional differences between the GC and HC groups (Fig. 1c), underscoring the potential of gut microbiota as biomarkers for GC. LEfSe analysis identified 31 taxa significantly associated with GC (Fig. 2a), contrasting with 18 taxa enriched in the HC group. These findings suggest that specific microbial signatures can serve as potential biomarkers for GC diagnosis. Random forest evaluated the potential ability of these specific gut microbiomes (Fig. 2b). Using the identified characteristic microbiota model, we predicted GC, achieving an AUC value of 0.81. Few studies have examined the diagnostic relevance of gut microbiota in GC; however, our published study appears to have demonstrated no superior ability to differentiate between the GC and HC groups (Zhang et al. 2021). The primary explanation for this phenomenon is the sample size. This study included a larger number of GC patients, nearly three times the number of newly diagnosed GC patients compared to our previous research. Increasing the sample size in gut microbiota studies allows our findings to better represent the true microbial characteristics of the population. Consequently, this study significantly improves the stability and resistance to interference of the data. The stability of these biomarkers over time and across different populations warrants further investigation.

## Correlation between tumor biomarkers and gut microbiota

While gut microbiota has been investigated as a biomarker for predicting immunotherapy response in GC (Gao et al. 2025), this study innovatively explores its integration with tumor biomarkers to enhance diagnostic accuracy in GC detection. Following the identification of differences in gut microbiota composition between GC patients and HC groups, we measured 10 tumor markers related to tumors in the collected GC patient cohort and a randomly collected validation group to determine whether GC patients have significantly elevated tumor markers. The analysis of tumor marker data in GC patients revealed an overall positivity rate of 60.78%. Approximately 40% of GC patients could not be diagnosed solely through elevated tumor markers. Among the tumor markers evaluated, CYFR21-1 exhibited the highest positivity rate in GC patients at 31.37%, indicating its limited efficacy for initial screening purposes. Liang Yao et al. (2016) identified notable limitations in the use of tumor markers as a primary screening method for GC.

Next, we attempted to use tumor marker expression levels for grouping, to determine the differences in gut microbiota composition between GCBP and GCNP. Interestingly, the analysis did not reveal significant differences in gut microbiota composition between those two groups. In lung cancer, Liu and his team found that gut bacterial communities in lung cancer patients with different positive tumor markers differ from those in healthy individuals (Liu et al. 2019). However, since there is no recognized tumor marker for GC, it is difficult to group individuals based on a single positive tumor marker. In this study, the positive group was defined as individuals exhibiting positivity for at least one of the 10 tumor markers. In these circumstances, no differences in composition were observed.

This lack of correlation indicates a complex interplay between tumor biomarkers and gut microbiota in GC, suggesting that tumor biomarkers alone may not fully capture the microbial alterations in GC patients. This finding highlights the importance of a multifaceted diagnostic approach that integrates gut microbiota profiles with tumor biomarkers to enhance diagnostic accuracy. Future studies should elucidate the underlying mechanisms driving the relationship between gut microbiota and tumor biomarkers in GC.

## Development of the diagnostic model

The relationship between tumor biomarkers and GC is complex; however, the sensitivity of tumor biomarkers in GC patients was greater than that observed in HCs. Thus, combining the characteristic gut microbiome in GC patients with tumor biomarkers may strengthen their potential diagnostic value. Our diagnostic model, which integrates gut microbiota profiles with traditional tumor biomarkers, demonstrated a high diagnostic performance, with an AUC value of 0.86 (Fig. 5b). This model was validated in an independent cohort, achieving a true positive rate of 0.84 (Fig. 5c), indicating its robustness and potential clinical utility. The gut microbiota-based model showed considerable accuracy (AUC of 0.81); however, its stability may not be sufficient for reliable clinical application. The integration of gut microbiota data with traditional tumor biomarkers improved the model’s performance and stability, suggesting that a combined approach may provide a more reliable diagnostic tool for GC. Although numerous new tumor markers for gastric cancer screening have been identified in recent years, such as urinary proteins (Shimura et al. 2020) and microRNAs (So et al. 2021), our combined model demonstrates excellent screening potential in both initial screening and validation.

## Clinical implications and future directions

Developing a non-invasive diagnostic model for GC based on gut microbiota represents a significant advancement in the field. This approach can reduce the reliance on invasive procedures such as gastroscopy, making GC screening more accessible and less burdensome for patients. The identification of specific microbial signatures associated with GC offers potential diagnostic biomarkers and insights into the mechanisms underlying GC pathogenesis.

Future research should validate these findings in larger and more diverse populations to ensure the robustness and generalizability of the diagnostic model. Longitudinal studies are necessary to evaluate the potential of gut microbiota profiles in monitoring disease progression and treatment response. Additionally, investigating the mechanistic interactions between gut microbiota and GC can reveal novel therapeutic targets and strategies.

Understanding the temporal stability of the identified microbial biomarkers is crucial. The gut microbiota is dynamic and can be influenced by various factors, including diet (Singh et al. 2017), lifestyle (Parizadeh and Arrieta 2023), and treatment (Chen et al. 2022b). Therefore, it is essential to determine the consistency of the identified biomarkers over time and under varying environmental conditions. Moreover, exploring the impact of different stages of GC and various treatment modalities on gut microbiota composition may yield additional insights into the potential role of gut microbiota in GC diagnosis and management.

This study has several limitations that should be acknowledged. First, the cross-sectional design limits the ability to infer causality between gut microbiota alterations and GC. Longitudinal studies are needed to establish temporal relationships and causal links. Second, while our model shows high diagnostic accuracy, it requires further validation in larger, independent cohorts. The study population was limited to a single geographic region, potentially impacting the generalizability of the results. Future studies should include diverse populations to confirm the broader applicability of the diagnostic model. Additionally, gut microbiota is influenced by various factors, including diet, lifestyle, and medication, which were not fully controlled in this study. These factors may potentially confound the observed associations between gut microbiota and GC.

## Data Availability

Sequences of this study have been uploaded on NCBI under BioProject ID PRJNA1135327 (available at https://dataview.ncbi.nlm.nih.gov/object/PRJNA1135327).
